# Counting the Population or Describing Society? A Comparison of English and Welsh and French Censuses

**DOI:** 10.1007/s10680-015-9372-y

**Published:** 2016-01-21

**Authors:** Ernestina Coast, Alex Fanghanel, Eva Lelièvre, Sara Randall

**Affiliations:** 1 Department of Social Policy, London School of Economics, Houghton Street, London, WC2A 2AE UK; 2 University of Bedfordshire, University Square, Luton, Bedfordshire LU1 3JU UK; 3 Institut National des Etudes Démographiques, 133, boulevard Davout, 75980 Paris Cédex 20, France; 4 Department of Anthropology, University College London, Gower Street, London, WC1E 6BT UK

**Keywords:** Household, Census, Survey, France, England, Comparability

## Abstract

Data collected at household level in censuses are used for a wide range of purposes including practical planning and academic analysis of changing social conditions. Comparability is a core demographic value, and to understand the limits of the comparability of census data across time and space, it is important to recognise if, how and why, concepts and definitions change between censuses. This paper examines definitions of the household in censuses in England and Wales (E&W) and France from 1960 to 2012 in order to investigate how census definitions have changed and to examine the drivers of such changes. Two research methods were used: (1) longitudinal analyses of census documentation since the 1960s and (2) in-depth interviews with key informants oriented around respondents’ roles in the collection and/or use of household data from censuses and surveys. We identify two contrasting national approaches to the data collection exercise that is called a census, which reflect political and institutional differences. These differences call into question the comparability of some aspects of census data across national boundaries, despite increased harmonisation of approaches to data collection. By comparing the evolution of the definitions of the “household” in censuses, we develop insight into the diversity of the priorities of census commissioners and designers, and consider the broader implications of this for producing comparable data.

## Introduction and Rationale: Census Data on Households

The effective use of man-power and the planning of land use, of housing, and of environmental, health and social services—all these must begin with the latest figures about the population both as it is now and as it will be in the future. (Hansard 1963)

C’est-à-dire que pour le recensement, l’objectif principal c’est de compter les personnes sur le territoire et les compter une seule fois, c’est pas forcément de reconstituer l’échelle pertinente de décision au sein d’un logement.^1^ (Civil servant, INSEE 2011)

National population censuses are the largest and most complex statistical operations conducted by states; 96 % of the world’s population was enumerated in the 2010 round of censuses (UNSD 2013a). The enumeration of population has been described as “the most visible, and arguably the most politically important, means by which states statistically depict collective identities” (Kertzer and Arel 2002, p. 3). There remain only a few countries which have never conducted a census (e.g. Eritrea, Western Sahara) (UNSD 2013b).

Census data are exploited by politicians, policy advisors, statisticians, local government, lobby groups and national and international organisations for the planning and allocation of resources. Data collected at household level in censuses are used for a wide range of purposes including: electoral arrangements; baseline data for planning local health and education services; to identify locations for future services and infrastructure; as a sampling frame for subsequent surveys; and for the estimation of vital statistics in the absence of other data. Historical and more contemporary census data have been extensively used in academic analyses from diverse disciplinary perspectives to understand different dimensions of social trends and transformations. For census data to be comparable and comprehensible across time and space, it is important to understand if, and how, concepts and definitions differ between censuses.

The costs of a census (time and financial) mean that “it is difficult to justify a census in strict economic terms” (Newell 1988, p. 15) but the “prestige” of doing a traditional census (Newell 1988) remains important for many countries, and can signal belonging to the international statistical community (Randall et al. 2015). A survey on census methods used in the 2010 round found that 85 % were using a “traditional” census for a total population count (all countries in Africa, North America, South America and Oceania), 10 % were using administrative registers and the remaining 5 % a different methodology altogether (UNSD 2013c).^2^ Debates, particularly in high income countries, about whether to replace the traditional census with administrative data are responding to a range of issues including cost, increasing availability of good administrative data and public concerns about privacy and potential data (mis-)use (Coleman 2013; Rowland 2003).

### Census Unit of Enumeration: Household

A key concept in censuses is the household, which is the unit for which much data are collected and analysed. In this paper we use the household as the lens through which we study census change over time. Interrogation of how census definitions evolve in the way that they do, and consideration of the way that they change, reveal some of the drivers behind census variations between countries. By comparing the way in which “households” have been defined in censuses we can gain an insight into what census commissioners and designers consider important—what their preoccupations and priorities are, what they think captures how people live, and, whether indeed, such representation can, or should seek to, reflect reality. The household enumeration unit excludes populations that live in institutional settings (military, hospitals, boarding schools, etc.), and we focus here only on non-institutional populations in England and Wales and in France.

Although some academic consideration has been given to the way in which “household” as a conceptual, statistical and analytical category has been used in social research in the global South (Bauman 1999; Beaman and Dillon 2010; Casimir and Tobi 2011; Randall et al. 2011), there remain few examinations of the ways in which the concept of “household” itself has evolved in Europe, and the implications for social science analyses (c.f.Hoffmeyer-Zlotnik and Warner 2008). Globally, UN agencies such as UNSD and UNFPA have been influential in the development and use of statistical definitions of the household. A 1959 UN document outlined:

A private household should preferably be defined as: (a) one person household: …(b) multi-person household: a group of two or more persons who combine to occupy the whole or part of a housing unit and to provide themselves with food or other essentials for living. The group may pool their incomes and have a common budget to a greater or lesser extent. The group may be composed of related persons only or of unrelated persons or of a combination of both, including boarders but excluding lodgers. (UN 1959, p. 74)

This UN definition remained largely unchanged for several decades. In 1980 the UN defined the household^3^ as:

1.223 The concept of “household” is **based on the arrangements made by persons, individually or in groups, for providing themselves with food or other essentials for living**….

1.226. Households usually occupy the whole, part of or more than one housing unit but they may also be found living in camps, boarding houses or hotels or as administrative personnel in institutions, or they may be homeless. Households consisting of extended families that make common provision for food or of potentially separate households with a common head, resulting from polygamous unions, or households with vacation or other second homes may occupy more than one housing unit. (UN 1980, p. 50)

In 1997 the part highlighted in bold was reiterated in a further UN document (UNSD 1997, p. 50).

The census household definition is driven by pressures of universality and the avoidance of double counting (Randall et al. 2015). Implicit in such enumeration units, and their definition, is a tension between the census’ mandate of accurately counting the population (once and only once) and providing information about people’s living arrangements.

Across Europe, there have been considerable societal shifts over the past five decades. The continuing reconfiguration of the nuclear family leads many people, children especially, to have two (or more) homes and belong to two (or more) households (ESRC 2006; Imbert et al. 2014; Stillwell et al. 2009). Highly mobile professionals may live parts of weeks or months in one place and parts in another. Changing social norms and socio-political institutions mean that levels, rates and ages of family formation, cohabitation, (re-)partnership, (re-)divorce and (re-)marriage have shifted substantially (Demey et al. 2013; Prioux et al. 2011; Rault 2012; Smallwood and Jefferies 2003; Stone et al. 2014). The emergence, for example, of living-apart-together (LAT) unions is one such example (Haskey 2005; Régnier-Loilier et al. 2009).

This paper compares and contrasts the approaches of censuses to reflect this changing society in two settings (England and Wales [E&W] and France) and addresses three research questions:

1. What are the implications of different national settings for census design and data collection?
2. In what ways, and why, does the unit of enumeration change over time and space?
3. What are the implications of national differences in census operations for understanding society, both within and across nations?

### International Comparisons: E&W and France

Our study design is one of international comparison, using the same research methods to address the same research questions in two different nation states (Hantrais 2009; Hantrais and Mangen 1996). E&W and France are high income European settings with similar demographic dynamics. Both have a long history of censuses, albeit with rather different historical roots for the collection of vital statistics. A comparative historical study of the emergence of demographic and vital statistics in England and France in the nineteenth century shows the “enthusiasm” of the English government for statistical data in order to better understand social problems such as poverty and public health; the French government was rather less enthusiastic (Schweber 2006).

In E&W the census is the responsibility of the Office for National Statistics (ONS), and in France the census is under the auspices of the National Institute of Statistics and Economic Studies (Institut National de la Statistique et des Études Économiques: INSEE). ONS is the statistical institute of E&W, headed by the National Statistician, accountable to the UK Government. Scotland and Northern Ireland have their own separate statistical offices, NRS and NISRA, respectively. ONS is responsible for the collection and publication of statistics, including the census, on economic, social and population data. The most significant recent change to the ONS has been its legislative independence in producing statistics, culminating in the establishment of the UK Statistics Authority (UKSA) in 2008. ONS funding is a part of, and is reported by, the UKSA, in turn funded by the UK Government. The ONS does not report to an individual Minister or Ministry.

INSEE is a Directorate General of the Ministry of the Economy, Finance, and Industry and receives its funding from the State’s general budget. INSEE organises the population census, produces the main indicators for the national economy (national accounts, consumer price index) and periodically conducts statistical surveys of households on specific topics (employment, living conditions, housing, health, etc.). Finally, INSEE also uses administrative data (civil registration records, annual income tax returns, etc.) for statistical purposes. There are therefore subtle but significant differences in the institutional arrangements between E&W and France for responsibility for the census (Table 1).

**Table 1:** Comparison of ONS and INSEE

	INSEE	ONS
Who funds	National government: direct via Ministry of the Economy, Finance and Industry	National government: indirect via UK Statistics Authority (UKSA)
Legislation	Census enshrined in law^a^	Legislative independence
Who answerable to	Ministry of the Economy, Finance and Industry	UKSA (independent of Government)
Staffed by	Civil servants	Civil servants
Census mission statement	“Chacun de nous compte^b”^	“Who we are. How we live. What we do”

^a^Law no. 2002-276 of February 27, 2002 (articles 156–158) establishes the principles for conducting the census and disseminating the annual official population of every commune. (http://www.insee.fr/en/methodes/default.asp?page=sources/ope-rp.htm)

^b^Trans: Each and everybody counts/is considered/is important. There is a pun on the word “compter” here

## Methods

Two research methods were used: (1) a review of census documentation (including census schedules, enumerators’ manuals, training materials, associated paperwork, and internal documentation) which was organised longitudinally to examine evolution since the 1960s and comparatively to examine similarities and differences between countries; (2) in-depth interviews (*n* = 24 UK, *n* = 25 France) conducted with key individuals situated at different places on the chain of data production (census designers, interviewers, statisticians, policy makers, diverse data users and academics) and oriented around respondents’ roles in the collection and/or use of household data. Discussion particularly focused on the way in which the “household” is defined and used in censuses and surveys. Recorded interviews were transcribed verbatim and coded using NVivo.^4^ After reading all the interviews multiple times the research team collaboratively developed a coding book with both descriptive and analytic codes. Some of the analytic codes focused on hypotheses which were developed before the research was undertaken, whereas others emerged inductively from the interview material and were the subject of considerable debate and elaboration by the research team. The data and analyses presented here are explicitly comparative, both over time (1960s–present) and space (E&W and France). For a detailed description of the broader project within which this work was conducted, including its ethical review, see www.householdsurvey.info.

We present our results and analyses thematically, integrating documentary and interview evidence throughout. We present all key terms or quotes in their original language, and provide authors’ translations into English in footnotes, in order to preserve the original nuanced vocabulary.

## Counting People or Understanding Their Living Arrangements?

I think the topic of household composition and household structure, and capturing it, is fundamental to how we understand relations across the life course and vital for policy and planning and vital for informing the assumptions of policy. (Academic, English University 2011)

A review of census documentation highlights significant, but subtle, changes in census household definitions over the past half century in England and Wales (Table 2). Analysis and interpretation of these definitions suggests three key analytic categories, present to varying degrees: sharing of space; sharing of food; and links with an address. Changing dominance of these categories over time can be understood as a reflection of changing social norms and living arrangements. We identify categories which our analyses suggest are the dominant or secondary theme(s) in each definition.

**Table 2:** Census definitions of the household, England and Wales, 1960–2011

Decade	E&W definitions	Analytic category (and relative importance)		
		Shared space	Shared food	Address
1960s	1961 One person living alone or a group of people living together, partaking of meals prepared together and benefiting from a common housekeeping. A person or persons living but not boarding with a household in a house, flat, etc., should be treated as a separate household. But a person living with a household who usually has at least one meal a day provided by that household while in residence is part of that household (breakfast counts as a meal for this purpose). A household must have exclusive use of at least one room. If two people share one room and do not have exclusive use of at least one other room, they should be treated as one household	Secondary	Dominant	Not present
	1966 Any group of persons, whether related or not, who live together and benefit from common housekeeping, or any person living alone who is responsible for providing his or her own meals. A person living but not taking meals with a private household was treated as a separate household, but if that person has at least one meal a day with the household he was regarded as part of that household. Breakfast counted as a meal for this purpose. By convention a household had to have at least one room. Two or more persons living in one room were regarded as one household regardless of whether or not they had meals together or shared common housekeeping	Secondary	Dominant	Not present
1970s	1971 Either one person living alone or a group of persons (who may or may not be related) living at the same address with common housekeeping. Persons staying temporarily with the household are included. A boarder having at least one meal a day with the household counts as a member of the household (breakfast counts as a meal for this purpose), but a lodger taking no meals with the main household counts as a separate one person household even if he shared the kitchen and the bathroom. A group of unrelated persons sharing a house or flat would count as one or as several households according to whether they maintained common housekeeping or provided their own meals separately	Secondary	Dominant	Secondary
1980s	1981 Either one person living alone or a group of people (who may or may not be related) living, or staying temporarily at the same address with common housekeeping. Enumerators were told to treat a group of people as a household if there was any regular arrangement to share at least one meal a day, breakfast counting as a meal, or if the occupants shared a common living or sitting room	Dominant	Dominant	Secondary
1990s	1991 Either one person living alone or a group of people (who may or may not be related) living or staying temporarily at the same address with common housekeeping. As in 1981, enumerators were instructed to treat a group of people as a household if there was any regular arrangement to share at least one meal (including breakfast) a day, or if the occupants shared a common living or sitting room	Dominant	Dominant	Secondary
2000s	2001 One person living alone or a group of people (not necessarily related) living at the same address with common housekeeping—that is, sharing either a living room or sitting room or at least one meal a day	Secondary	Secondary	Dominant
2010s	2011 One person living alone or a group of people (not necessarily related) living at the same address who share cooking facilities and share a living room or sitting room or dining area	Secondary	Not present (only facilities not food)	Dominant

*Sources* General Register Office (1962, p. 2), General Register Office (1968, p. 11), Office of Population Censuses and Surveys (1979, p. 9), Office of Population Censuses and Surveys (1981, p. 6), OPCS (1992, p. 10–11), ONS (2004, p. 34), ONS (2013, p. 20)

In 1961, common housekeeping is central to the definition. In English the words “household” and “housekeeping” suggest not only the importance of the dwelling house but also of the relationships within that physical structure; the holding or keeping of ties between individuals—whether related or not—within a dwelling. In 1961, shared meals and the exclusive use of a room as well as the need for shared housekeeping make the household. However, no indication of what should be understood by common housekeeping is given. The requirement that people within a household must have exclusive use of a room offers a spatial dimension to the definition. This aspect appears in subsequent household definitions, but, crucially, the exclusivity of the room requirement is eventually removed.

In 1966, common housekeeping and shared meals together define the people in a household, although different households cannot share one room regardless of whether they eat meals together or not. In 1966 further specificity was introduced, focusing on breakfast, possibly introduced in order to counter ambiguity about what constitutes a meal as well as reflecting changes in lifestyles.

This 1966 definition was used in a one-off trial 5-year census of a 10 % sample of the population of E&W. Hansard (the parliamentary record) reports suggest that this interim census survey was required to accommodate contemporary “rapid change and development” and to plan resource allocations indicating the importance placed in E&W censuses of reflecting society (Hansard 1963). The complication of not accurately reflecting poorer single-person households which was a consequence of the exclusive room requirement in 1961 and 1966 was addressed in part by changes to the definition in 1971.

While the common housekeeping requirement (still undefined) remained, in 1971 far greater emphasis was placed on the requirement to share a meal a day for inclusion in a household unit for apparently unrelated people (boarders and lodgers). The definition requires that the household must share common housekeeping, and that there is a subsequent meal requirement. The one household/several household distinction turns on whether co-residents share either common housekeeping or meals. No definition is given of “common housekeeping”, but it evidently excludes the sharing of meals which, without common housekeeping, is sufficient for a group of unrelated persons to count as a household. This part of the definition is in tension with the earlier section where both requirements appear to be necessary. These nuances introduce an element of hierarchy: a lodger/boarder is attached to an existing household if she/he fulfils the criteria, but the “main” household already exists. By contrast, a flat-share is a collection of equals without there being a “main” household that would otherwise exist. The 1971 definition of the household refers to address for the first time but excludes reference to any spatial requirements/rooms—whether shared or exclusive for identifying households. Consumption practices and arrangements were key to the definition in 1971. In earlier E&W definitions there is the assumption that people live together behind the same front door—although this is not outlined explicitly until 1971 when “living at the same address” is integrated into the definition.

Spatial criteria return in 1981: a household must live at an address and share common housekeeping (not defined). The shared meal for identification of a household endures, but is no longer essential. In 1981, for a household to be identified, an alternative to sharing meals is sharing a living room. This has changed from exclusive use of a room required in 1961 and 1966. Sharing a living room is sufficient for people who live together (e.g. group of friends) but do not eat together to be counted as a household—possibly reflecting a widespread increase in house/flat sharing between students or young unrelated adults. The sharing of a meal means that people who lodge with a household and whose meals are provided count as part of the household even if they do not use the common rooms. This will tend to capture a greater number of people within one household. One reading of this definition suggests that shared housekeeping could include either the shared room or the shared meal, but this only becomes explicit in 1991 where the definition clarifies that common housekeeping means either a shared meal or a shared common space. Thus, as long as the person or persons live at the same address and share a meal or a common room, they count as a household. This 1991 definition is almost exactly the same as the 2001 definition.

In 2011, three further subtle changes appear; the concept of “common housekeeping” disappears (possibly reflecting the fact that it was always rather unclear), and the requirement for the meal to be shared is replaced by the requirement to share cooking facilities and to share a living room or sitting room, or dining area. The household members must still all live at the same address. The sharing of cooking facilities as opposed to meals takes an element of the consumption requirement out of the definition; it is no longer necessary that a household is nourished from the same larder; they do not need to eat at the same time or eat the same thing. This reflects the fact that fewer household members (whether related or not) eat together in England and Wales, and better reflects the increase in unrelated individuals—often students, young people or migrant workers—who share dwellings, particularly in urban centres, because of increased housing costs.

Through these nuanced changes in wording, census household definitions in E&W were attempting to reflect changes in the ways that people manage their lives in terms of co-residence, eating and managing living space. There will always be arrangements that are difficult to capture, but the essential point is that the definitions are trying to pre-empt major trends. Such subtle definitions also require trained census-enumerators for clarifications: the general population which the census enumerates, are not all able to grasp these subtleties.

By contrast, such nuanced changes are totally absent in France. Differences between France and E&W emerge on a number of fronts. Definition changes in E&W focused on identifying whether there should be one or several households behind the front door. In France, the opposite concern is paramount:

Je pense que quantitativement l’attention se porte d’ailleurs plutôt sur les ménages qui sont sur plusieurs logements que le même logement avec plusieurs ménages.^5^ (Academic Data Analyst, 2011)

In France until 1954 there were some ambiguities in household definition, but post-1954 the idea of “ménage-logement” (household = dwelling)^6^ became fixed, defined as:

l’ensemble des personnes, quels que soit les liens qui les unissent, qui habitent une unité d’habitation privée, c’est-à-dire, un local séparé et indépendant. (INSEE 1968)^7^

The French census is recorded on a de jure basis encompassing those in their “résidence principale*”* (main residence) whether or not they were present on census night. This is because the purpose of the French census is an integral part of commune administration. Finally, and in contrast to E&W, French census forms have been self-completed since 1881. This demands a simplified and straightforward unit—“le logement”—within which everybody is counted.

In the instructions accompanying the self-completion forms (bulletin) for the French census, there has been a clear distinction between the family and the household, the latter being: “unrelated individuals usually living in this place, whether related or not, need to be listed and fill a form” (author translation). The unambiguous definition of “ménage-logement” was set out in the 1954 census, and by 1962 questions about family ties were excluded from the bulletin. However, in 1968, the notion of “résidence principale” was introduced, corresponding to permanently occupied, fixed dwellings. This had the effect of excluding individuals who live in barges, caravans, etc., which, until then, had been included as households. Since 1968 people in such mobile accommodation have been enumerated separately.^8^ The implication is that, if they are not in a fixed house with an address, they do not constitute a household.

In 2004 the French census was transformed into a rolling census (Desplanques 2009) and the definition changed from “résidence principale” (principal residence) to “résidence habituelle” (usual residence) defined as “the place where you live the longest during the year”. This means, for example, that students living away from the parental home for much of the year or older people who live in residential care accommodation but still own their home, are now enumerated where they live for most of the time. This has ramifications for household structure and size. However, these changes have not been made in response to attempts to better represent living arrangements but more to distinguish between the fiscal unit (“foyer fiscal”) which identifies people declaring a joint revenue but who live separately, and the actual local residents who use local authority services and amenities in the commune where they spend most of the year.

The French census definition of the household (ménage-logement) itself remains identical throughout the whole half century despite broadly similar transformations in living arrangements to those occurring in Britain. This definitional stability in France is because of constraints posed by the administrative and legal obligations behind the French census and the fact that the key unit in the French census is the logement; the individuals within that logement need to be enumerated as individuals and do not need to be placed into households representing some sort of basic economic unit in society.

“…dans le recensement français nous n’avons rien sur les revenus. Donc l’idée de partager le même budget, de vivre sur le même revenu, on ne peut pas l’appliquer au recensement—et les gens du recensement ne veulent pas même qu’on pose une question sur le revenu, même qu’on aborde le sujet des revenus, en disant quand même que le recensement c’est pour compter des personnes, donc il faut que les gens répondent le mieux possible au recensement et donc n’abordons pas les sujets qui pourraient être jugés indiscrets, qui pourraient bref fâcher—donc […] on est resté sur la notion de ménage—logement.”^9^ (France: INSEE Survey designer, 2011)

Thus INSEE interprets the UN guidance to take into account the ways people organise themselves, in terms of budget sharing, and not in terms of common provisioning as is done by ONS. But, because collecting information on budget sharing is incompatible with the census ideology, in France the logement (i.e. the physical dwelling) became, and defines, the ménage (household).

## Managing Fieldwork (Terrain)

ONS and INSEE both have to deal with the practicalities of census data collection. In E&W in 2011, census forms were posted to the occupants of each registered address in the Postal Address File:

… it’s borne out of the practicality really of wanting to be able to enumerate these people and because it is not immediately obvious. It is a front door, the postman’s putting something through the door, so we’ve got no idea whether we’ve got 5 rooms of people who count themselves individually or not, so we can deliver one questionnaire and we can make clear on there if you all share a kitchen and some living space essentially, a cooking space and living space is what we’re after, if you share those facilities we’re counting you as one household. (E&W: ONS Survey designer, 2011)

A key driver for the change in definition of the household in 2011 was the way in which the census was administered:

In 2011 because the questionnaires were posted out and posted back, so we weren’t going to see people, that had to change, but part of that came from the experience in 2001 where in the 2001 census the definition was broadly comparable with that which had gone previously so it was a group of people living together sharing common housekeeping was the term, and the questionnaires were hand delivered so if the field staff made contact they could explain that but what we found increasingly was that our field staff who were then supposed to be making that judgement, making that decision, weren’t making contact [with respondents], particularly in London and other cities. (E&W: ONS Survey designer, 2011)

Changing technology and resource constraints have reduced the use of enumerator-administered census forms, meaning that the definition had to be easier to interpret by respondents:

It was also then clearer when we didn’t make contact with the householders—which we weren’t doing—in that we were posting the questionnaires out, for them to understand what we were looking for, so we took away the social element of it and made it physical for practical reasons. (ONS Survey designer, 2011)

French census forms have been self-completed since 1881, and enumerators only distribute the forms and retrieve them (“le dépôt-retrait”).

En France on n’a pas de répertoire des personnes –donc pas de répertoire des ménages- on n’a pas le droit de créer ce type de répertoire […] on avait par contre, une base de logements, c’est moins sensible politiquement et donc on a considéré qu’on partait de l’unité observable, le logement […] et on a assimilé un logement égale un ménage.”^10^ (France: INSEE civil servant, 2011)

“…en fait l’INSEE tire finalement essentiellement des échantillons de logements, qui vont être des résidences principales pour les personnes d’un même ménage et c’est là qu’on va trouver nos enquêtés, c’est une porte d’entrée.”^11^ (France: INSEE-trained statistician, 2011)

In France the census enumerator is given a list of logements as recorded in the Répertoire d’Immeubles Localisés (RIL). This register which is maintained and up-dated by each municipality (or commune) gives the number of accommodation units/flats (logement) at each address. The enumerator must distribute a Bulletin Logement to each dwelling (logement) on the list and leave the correct number of individual questionnaires (Bulletins individuels) for the occupants. All usual occupants must fill an individual bulletin and be listed on the Bulletin Logement.

## The Census as a Reflection of Society and Daily Life: Perspectives from Key Informants

Analysis of E&W census documentation suggests that the household definitions generated by ONS are making substantial efforts to accommodate and represent changing social realities. In fact there was a very substantial shift between 2001 and 2011 motivated by two, very different drivers: practical, methodological considerations, and an attempt to reflect contemporary changes in the organisation of everyday life. The nuanced changes to the definition of the household over the past 50 years were generated by the second of these drivers, whereas getting rid of common housekeeping and the shared meal in 2011 was generated by the practicalities of 2011 census data collection although it does also reflect changing trends in meal consumption.

Two UK-based University academics who not only use household data in their research but have also worked with ONS and other international statistics agencies to analyse census data suggest that the impetus behind past changes to definitions has been primarily to reflect changes in social life:

The household, in that respect, is defined as a sort of dwelling space where you’re the only person in that space, typically a flat. The rules have changed as to how you’re supposed to define household. In the past they’ve talked about…everyone in the household sitting together [.] down together to eat a communal meal. Um that’s sort of…been removed. I think I’m getting this the right way around, I hope so, as that’s simply socially become a less common occurrence. (E&W: Academic Data Analyst, 2011)

The questions they ask, how do you identify several households within the same dwelling, for example I remember a traditional definition of household in Britain was… people who share, cooking facilities, I think that is the latest definition, because it used to be people who share a meal, and then they realised that nobody shares meals nowadays. (E&W: Academic working on households, 2011)

This is very different from INSEE where the census prioritises very different dimensions: counting individuals and providing legal population counts is what is really important, not how they organise their lives (see quotes at the beginning of the paper) even though census data are used to publish statistics on commune inhabitants and their characteristics: composition by sex and age, occupation, housing conditions, modes of cohabitation, etc.

In contrast both British academics suggest that the definition of the household had changed—and that the shared meal requirement, in particular, had disappeared—because of ONS’s attempts to define a household in such a way as that it continues to reflect the reality of the majority of the population to whom the definition is applied.

[to] share the kitchen, that’s quite standard, and what we’ve tried to do is make sure…Those people could go their lives never talking to each other, they just happen to share a cooker, so we count those as separate households, which is why we started saying you have share a lounge or a dining room, there has to be some kind of social space that you’re sharing for us to count you as household. (E&W: ONS Survey Designer, 2011)

The importance of the social, in what makes a household—the “hold” of the house—becomes clear and accounts for the “and” in the 2011 definition. The imperative to create a household definition which reflects the changing way in which people live appears to be the rationale for this historical series of subtle changes.

In France, things are very different: the census “household” is not regarded as a conceptually strong entity for data producers and consumers. From our interviews with data users the term “ménage” has different meanings according to data sources and institutions using them:

On aura en fait trois unités statistiques qui ressemblent au ménage et qui sont distinctes, la première qui est liée à la cohabitation dans le logement, c’est le ménage, la seconde qui est couverte par les droits sociaux…, c’est l’assuré social et ses ayants droit …la troisième c’est le foyer fiscal parce qu’en France l’imposition est collective qui rassemble les parents et les enfants…y compris les enfants majeurs et ne vivant pas avec leurs parents, peuvent joindre leur déclaration de revenus à celles de leurs parents…pour des raisons avant tout d’optimisation fiscale.^12^ (France: Academic Data Analyst, 2011)

Three types of “ménages” are identified each corresponding to institutions: administrative data producers, social benefits providers and fiscal collectors. Only the first of these is the census household, and it encompasses those people who live behind the door of a “logement*”* with no interest in how they organise their daily lives: thus it is clear that the French census household cannot really be compared to the E&W census household, but this is justified by the potential triangulation of French data sources.

L’objectif principal du recensement c’est compter et donner des informations qui intéressent les communes […] Donc par exemple dans le recensement on ne fait pas la différence entre une famille recomposée et une famille traditionnelle, les liens sont donnés mais comme c’est du traitement de masse ils ne sont pas exploités.”^13^ (France: Senior civil servant, INSEE, 2011)

French data producers are constrained by the need to ensure that the final counts are tenable, and that the data collected are, at the national level, compatible with other sources both in terms of housing stock and fiscal declarations.

Mais l’estimation que l’on donne de la population en France c’est pas le recensement tout seul. C’est un mélange entre l’observation de la collecte et l’utilisation des données de la taxe d’habitation.^14^ (France: INSEE Civil servant, 2011)

La loi permet l’appariement, l’INSEE reçoit des données de toutes les autres administrations, y compris fiscales mais uniquement à des fins statistiques.^15^ (France: Academic Data Analyst, 2011)

In such circumstances any attention given to how the “household” represents people’s living arrangements is secondary.

## Household Heads and Reference Person

Although the household is supposedly a statistical unit which is independent from “family”, in reality the two are closely intertwined and the presence of a “family” in a household is an important element in many analyses. Rules about who was referred to as “household head” and subsequently “reference person” are illuminating. The notion of a “head” suggests traditional patriarchal social structure and authority based on age and sex, although these were not enshrined specifically in the definition. In contemporary society such a concept seems archaic and inappropriate. Yet, for data collection and analysis of household form and structure it is useful to have one individual to whom others can be linked.

[In E&W] the head of household is the first person on the form who was (1) aged 16 years or over, and (2) usually resident at the address of enumeration (…) The head of family or family unit is taken to be the head of household if the family unit contains the head of household. (OPCS 1992, p. 32)

It was only in 2001 that E&W adopted the concept of household reference person: “for a person living alone, it follows that this person is the Household Reference Person (HRP). If the household contains only one family (with or without ungrouped individuals), the HRP is the same as the Family Reference Person (FRP). If there is more than one family in the household, the HRP is chosen from among the FRPs using the same criteria as for choosing the FRP economic activity, then age, then order on the form. If there is no family, the HRP is chosen from the individuals using the same criteria” (ONS 2004). As the new designation shows, there is a systematic priority given to the family within the household. It is only in 2011, that the “hold” supplants the family with a new procedure: *“*A Householder (or joint householder) is the person resident or present at the address who: (a) owns/rents the accommodation and/or is responsible for paying the household bills and expenses” (ONS 2013)

In France, no precise definition of the head of the household was ever given on the bulletins or in the census instructions (Courson 1982). Up to and including the 1975 census a “household head” was designated for each household in France and either this person was self-declared or was ascribed this role at the moment of the census; theirs was the name in the first position of the household membership list. Courson suggests that the recorded household head reflected implicitly a combination of “traditional” characteristics which justified this position. After the social unrest of 1968 it became socially problematic to use the notion of “household head” so the 1982 French census replaced household head with “reference person”, that is, “the person who without regard to gender is the oldest economically active or the oldest person in the household” (INSEE 2013). Thus there was an attempt in France to reconfigure the language around the census household according to social trends. Despite this, because a majority of households were constituted of families, there was probably little change in the designated person. Indeed, the instructions given for non-familial households continue to base criteria for identifying reference persons on age, activity and gender:

S’il n’y a aucune famille dans le ménage, on retient comme personne de référence du ménage la plus âgée des personnes actives du ménage ou, s’il n’y a aucun actif dans le ménage, la personne la plus âgée du ménage. S’il y a une famille dans le ménage, on retient comme personne de référence du ménage l’homme (l’adulte de sexe masculin) dans le cas d’un couple ou la personne adulte sans conjoint dans le cas d’une famille monoparentale.^16^

In E&W analysis of the structure within the household—once it has been identified—focuses on the relationships between all members captured in a household grid. In France such data are collected but not coded or analysed because understanding and representing how people organise their lives is not an aim of the census.

## Institutional Drivers

Census requirements that every individual be counted once and once only, distorts the picture in both France and E&W because neither approach is very good at capturing the increasingly complex and mobile living arrangements of the twenty-first century.

Both E&W and French censuses rely on an apparently common unit of enumeration (household/ménage), the definition of which is inspired by the UN directives and accommodated by their national statistical organisations. The interplay between the objectives of the census and the constraints and practicalities of data collection lead to different strategies and practices in the conduct of the census, within and between the two settings. In the previous section we reflected on social and practical drivers of these changes. We suggested that in E&W, priority is given to an attempt to capture “Who we are. How we live. What we do” by adapting the household definition to reflect social change, whereas the French aim of an exhaustive count of the population (“each and everybody counts”) rather than analysing social change is reflected in the continuity of household definition since the 1960s.

But how far do institutional drivers, meaning administrative pressures, also influence E&W statisticians in their design and use of household definitions? Is reflecting real life really as paramount as first appears, or do other data constraints also figure in their strategies? The 2011 E&W census definition no longer includes “common housekeeping”. Is this because common housekeeping is an ambiguous term in 2011? Or, are the practicalities of using the definitions in data collection being prioritised?

Because of the changes that we’ve made of [sic] our definitions are for practical reasons, that’s the—it’s not because, it’s not because, the way people are living is changing but that’s not really why we’re changing our definitions, we’re changing our definitions because we have to make them useful to us to actually be able to count people, that’s really been the driver. (E&W: ONS Survey designer, 2011)

Population transformations such as urbanisation and migration, and changing ways of organising domestic lives—eating meals separately, strangers sharing accommodation—certainly play a part in understanding the evolution of this definition, but also, as ONS suggest here, the dominant driver is the need to make definitions which actually facilitate counting people. When understood against this background, the definition of the E&W household looks like a statistical unit primarily to satisfy census requirements of universal coverage without double counting, rather than one which attempts to reflect changing social reality.

On the other hand, the principles defining the objectives of the French census are imposed by law (see Law no. 2002-276 of February 27, 2002 (articles 156–158)): establishing the official population of each commune comes under the authority of the State. Particular attention is given to the equality of treatment of all municipalities across all the national territory, because of the importance of these population counts for the establishment of the electoral constituencies and the budget of each local authority (Godinot 2005). The census is a legally embedded operation, under the authority of a Directorate General of the Ministry of the Economy, Finance, and Industry (INSEE):

La collecte est déléguée aux communes sous la tutelle scientifique et le contrôle de l’Insee. C’est l’Insee qui identifie le calcul des populations légales […] il y a des enjeux très importants pour les communes puisque les subventions allouées dépendent de la taille de la commune.^17^ (France, INSEE Civil servant, 2011)

As our interviews testify, counting people and providing the legal population counts of municipalities is the priority of the French census. Due to these institutional drivers, there is little flexibility in, nor is there the capacity for evolution of, the household definition in the census which is tied to strong legal obligations. Since INSEE must provide the municipalities’ legal population counts, there is a strong imperative to avoid double counting of individuals; this has driven the changes in French census data collection since 2004.

Before 2004, when the French census covered everywhere in a single round, two population counts were produced according to whether “double counts” were included—individuals possibly surveyed in two places (students in their hall of residences and at their parents’, for example). Municipalities’ legal population counts included “double counts”, and the total population of France was computed without “double counts”. Since 2004, the census is based on an annual data collection, successively surveying all the municipal territories over a five-year period (a rolling census). Five census surveys are used to produce the census results in the middle year (2004–2008 for the census population of 2006). In order to better evaluate local population counts, the calculation of the legal population count also relies on fiscal data even though taxation is not individually based in France. Thus the census is not a data collection exercise in isolation trying to get a snapshot of the social situation of the country but a negotiated solution to an administrative problem.

## Discussion

We thus observe two totally different national approaches to the data collection exercise that is called a census: these approaches reflect profound political and institutional differences and have epistemological ramifications which call into question the issue of comparability of some aspects of census data across national boundaries. In countries where the census is an integral part of the administrative and legal framework those dimensions will dominate the ways in which the data are collected, classified and potentially analysed. Thus, in France, there is little pressure for the units of census data collection to reflect societal-level changes in living arrangements unless they influence the “lodgement”. In such circumstances where people are not encompassed within the critical administrative unit [in France the “logement”] they will be excluded. Therefore, in France because mobile housing (barges, boats and caravans) cannot be permanently ascribed to a geographically defined municipality, people living in such accommodation have been excluded from the census of ordinary households since 1968 and enumerated separately. In the UK where the census has no administrative association such people and their mobile accommodation should be included because they, and their housing/social needs, are required to be represented for planning purposes and for analytic completeness.

UN guidelines for census data collection seem to assume a lack of administrative constraints on who is included, excluded and how they organise their living arrangements. For example the UN definition (1980, p. 4 above) talks about homeless people living in households—but in France without a “logement” such people cannot constitute a household.

Thus, worldwide, it is necessary to examine how censuses are related to national administration. The institutional embeddedness of censuses constitutes part of the explanation of the lack of adaptation to social trends in living arrangements. However, there are two interpretations of the subtle changes in definition observed in E&W censuses: either they are motivated by the need to change the definitions in order to best fulfil the fundamental census requirements of enumerating every single person once and once only, or they are motivated by pressure to be able to undertake meaningful analysis of social trends in living arrangements. Although one might like to interpret them as the latter, the former is the more realistic endeavour and this is reinforced by the changes introduced in the 2011 census. These changes make it clear that the priority is being able to collect the data and enumerate the absolute numbers—not represent accurately the complex living arrangements going on behind the door; managing the fieldwork to generate accurate data is paramount. Having an enumerator-assisted census allows subtle definitions to be applied and changed, compared to the simplicity required by self-administered questionnaires whether they are delivered, posted or completed on the internet.

This should not really be surprising. The census is, after all, a rather crude accounting tool. On a huge scale and undertaken very rapidly, with lots of negotiations about what questions can be included or are dropped, the census is never, and cannot provide, a sophisticated analysis of complex living arrangements. This should be the role of sample surveys which have the potential to represent a much wider range of complex social realities, including multiple household affiliations.

When understood against this background, the definition of the census household looks like a statistical unit only, rather than one which reflects reality. However, if, as in E&W, censuses move away from being administered or facilitated by trained enumerators towards self-completion by individuals, not surprisingly household definitions need to relate relatively closely to what people filling in the questionnaire themselves recognise as such, in order to get relatively complete data. Censuses, and definitions associated with them, must navigate this complexity. The negotiation of this tension, slowly over the past 50 years, against a background of rapid socio-economic and demographic change in England and Wales is reflected in these household definitions.

Constrained by its administrative role and self-completion since the nineteenth century, the overall French definition of the census household has not altered significantly over the past 50 years. Nevertheless, there are subtle changes at the margins reflecting social change: the move from household head to reference person and insertion of first “résidence principale” (1968) and then “résidence habituelle” (2003). However, the major changes have been implemented not for the census but for household surveys—which are a much more powerful and sensitive tool for representing social life, living arrangements and transformations therein.

Given that, according to a high-level INSEE civil servant “la définition du recensement est artificielle”^18^ and “dans le recensement on ne peut pas [modifier les modes de comptage] à cause de ces contraintes sur la définition de la population légale”,^19^ the effort of introducing changes to take into account individuals who belong to several households or individuals co-residing but having separate budgets, led INSEE to introduce another household definition which is to be used in all household surveys but not the census. It is based on “unités de vie” (living units) whose members can co-reside or be spread over several dwellings. At the same time one dwelling may house several “unités de vie”. Members of the same “unité de vie” share living expenses together. They were introduced to identify the economic decisional node within the dwellings. INSEE only envisaged the budget-sharing aspect of living arrangements suggested by the UN definition and not the meal-sharing or “cooking-pot” aspects, long adopted by the UK census designers, although one could argue that the meal sharing is, in fact, a proxy for the economic unit of consumption. Distinguishing the “unités de vie” proves cumbersome to collect, subject to enumerators’ perceptions and not absolutely obvious to respondents, as our interviews both of INSEE interviewers and survey designers show. Nevertheless, they then become a unit that moves much closer to the household that the E&W census—and British sample surveys—are trying to capture.

## Conclusion

Do these differences matter? In both countries the main aim of the census is to produce a total population count, with the potential to disaggregate at the local level. Our comparative analyses show that country-level social, practical and institutional drivers impact the fundamental social unit that is enumerated in the census. Recent harmonisation of statistical concepts across the EU area aims to produce “better” survey data which are comparable and reliable (Keuning and Morais 2010; Poulain et al. 2006; THESIM 2006). The census however is a data collection tool that is, and probably should remain, outside this harmonisation approach. The practical constraints of a census—enumerating people once and once only—mean that it is ultimately an accounting tool rather than a tool for really understanding social life. People do not organise their lives in ways that are easily captured in one place at one time. By its very nature, the census is a highly constrained attempt at representing society.

Our comparison of E&W and France highlights that the principal constraint of avoiding double counting in a census inevitably means that major compromises have to be made in the ability of the data to represent contemporary realities. The aim of a total enumeration is probably achieved although this may be more accurate for France than E&W because of the triangulation of a number of data sources. Those who are excluded from the E&W census are probably not omitted because of the nuances of household definitions but either because they want to remain invisible or they are socially and spatially marginalised and thus missed by the whole census operation; definitional changes are not going to increase contact with, and data from, such people.

We suggest that household definitions are and always will be imperfect and they will be more imperfect in the census than in surveys. Given the double obligation of being a useable meaningful term in census collection to data analysts and to people filling in the census form, the census household definition, whether in E&W or in France, will never be a subtle tool for capturing transformations in social life in the smallest units of society. It is critically important not to make data do more than they can do, and it is essential that researchers are aware that, despite standardisation and harmonisation, the term “household” may mean different things in different contexts and is not strictly comparable. For non-academics who use or cite census data, care needs to be taken in translating from a commonly used term in everyday language to the same term used in data collection exercises.

## Footnotes

1 Translation: For the census the principal aim is to count all the people in the land and to count them once only, it’s not about finding out about the relevant level of decision making within a dwelling.

2 For example, Germany uses a combination of traditional housing census, sample survey and register-based census (Valente 2010).

3 The sub-heading “Household” included “[or family]”, underlining the mutability of these concepts.

4 Anonymised transcripts of the UK interviews have been deposited in the ESRC data archive and can be found at: http://store.data-archive.ac.uk/store/collaborativeCollectionEdit.jsp?collectionPID=archive:730&tabbedContext=collCollection&collectionTitle=null.

5 Trans: I think that quantitatively, our attention is focused more on households spread across several logements/dwellings than on several households in one logement/dwelling.

6 The “logement” or dwelling in France cannot be translated by “address”: in France a large house split into flats will all have the same address although each flat is a different logement. The best translation seems to be dwelling which represents all those people who reside behind a front door. Henceforth we will use logement, or “dwelling” as the translation of logement.

7 Translation: All those individuals, whatever the links that have brought them together, who live in one private residential unit, that is to say a dwelling which is separate and independent.

8 See INSEE document: http://www.insee.fr/fr/ppp/sommaire/imeths01c.pdf.

9 Translation: in the French census we collect nothing about income. Thus the notion of budget sharing, of living off the same income, we cannot use it in the census—and the census people would not even agree to it, not even agree that a hint be made about it; they would say that, after all, the census is made to count people therefore in order for them to answer reliably, let’s not talk about touchy subjects which could antagonize them—thus […] we have remained with the concept of “ménage-logement”.

10 Translation: In France we have no population register (not even postal); therefore there is no register of households—we do not have the legal right to create such a register […] on the other hand we do have a housing register [logements] that is less politically sensitive and so we start from the observable unit, the dwelling [logement] and we assume that a “logement” equals a household.

11 Translation: In fact INSEE effectively draws their samples from “logements” which will be the main residence for people of the same household and that’s where we find our respondents—that’s the entry point.

12 Translation: In fact we have three statistical entities which resemble a household but are distinct, the first is defined by cohabitation in the same dwelling, it is the “ménage”, the second one is defined by social entitlement […], it refers to the person paying/receiving social security contributions/benefits and the his/her eligible dependents (partner, children etc.) […] the third one is the fiscal unit because in France taxation is collective and can group parents and children […] even adult children who do not live with their parents can attach their income revenue declaration to those of their parents […] in order to minimise their taxes.

13 Translation: The main aim of the census is to count and give relevant information to the municipalities […] For example in the census, no difference is made between a family with stepchildren and a traditional family, the family ties are declared but since it is mass processed, these are not analysed nor coded.

14 Translation: Nevertheless the estimates given of the total population and local populations in France do not come from the census only. It is a mix between the census enumeration matched with the data from the council tax.

15 Translation: INSEE has a legal right to match data, INSEE receives data from all the other administrative bodies, even the Inland Revenue, but purely for statistical purposes.

16 Translation: If there is no family within the “ménage”, the reference person is set to be the oldest of the occupied persons in the household, if no one is employed in the household, the oldest. If there is a family identified in the household, the reference person is set to be the adult male when there is a couple or the single adult in case of a one parent family. Note no. 52 des Résultats du recensement général de la population de 1982.

17 Translation: Data collection is now delegated to the municipalities under the scientific and legal control of INSEE. INSEE is in charge of the calculation of the legal population…a lot is at stake for the municipalities because the amount of their national subsidies rely on the municipality legal size.

18 Translation: The census definition is artificial.

19 Translation: In the census we cannot [modify the modes of enumeration] because of the constraints around the definition of the legal population.

## References

[CR1] Bauman KJ (1999). Shifting family definitions: The effect of cohabitation and other nonfamily household relationships of measures of poverty. Demography.

[CR2] Beaman L, Dillon A (2010). Do household definitions matter in survey design? Results from a randomized survey experiment in mali. IFPRI Discussion Paper.

[CR3] Casimir GJ, Tobi H (2011). Defining and using the concept of the household: A systematic review. International Journal of Consumer Studies.

[CR4] Coleman D (2013). The twilight of the census. Population and Development Review.

[CR5] Courson J-P (1982). Les ménages n’auront plus de chef. Économie et statistique.

[CR6] Demey D, Berrington A, Evandrou M, Falkingham J (2013). Pathways into living alone in mid-life: diversity and policy implications. Advances in Life Course Research.

[CR7] Desplanques G (2009). Strengths and uncertainties of the French annual census surveys. Population (English Edition).

[CR8] ESRC. (2006). *Changing household and family structures and complex living arrangements.* Paper presented at the ESRC Seminar Series: Mapping the public policy landscape.

[CR9] General Register Office (1962). Census 1961 England and Wales population dwellings households.

[CR10] General Register Office (1968). Sample census 1966 England and Wales household composition tables.

[CR11] Godinot, A. (2005). Pour comprendre le recensement de la population. *Insee Méthodes*. http://www.insee.fr/fr/publications-et-services/sommaire.asp?reg_id=0&ref_id=IMETHS01.

[CR12] Hansard. (1963). *House of commons debate* (Vol. 686 cc850-3).

[CR13] Hantrais L (2009). International comparative research: Theory, methods and practice.

[CR14] Hantrais L, Mangen SP (1996). Cross national research methods.

[CR15] Haskey J (2005). Living arrangements in contemporary Britain: having a partner who usually lives elsewhere and living apart together (LAT). Population Trends.

[CR16] Hoffmeyer-Zlotnik, J. H. P., & Warner, U. (2008). Private household concepts and their operationalisation in national and international surveys. In *Survey methodology* (Vol. 1). Mannheim: GESIS-XUME.

[CR17] Imbert, C., Deschamps, G., Lelièvre, E., & Bonvalet, C. (2014). *Vivre dans deux logements: surtout avant et après la vie active*. Institut National d’Études Démographiques (INED).

[CR18] INSEE (1968). Recensement général de la population de 1962: ménages—familles.

[CR19] INSEE (2013). http://www.insee.fr/en/methodes/default.asp?page=definitions/personne-reference-menage.htm. Accessed 17 October 2013.

[CR20] Kertzer DI, Arel D (2002). Census and identity: The politics of race, ethnicity, and language in national censuses.

[CR21] Keuning, S., & Morais, A. (2010). European statistical harmonisation and improvements to serve the needs of the European Economic and Monetary Union. *ICF Bulletin*, pp. 134–138.

[CR22] Newell C (1988). Methods and models in demography.

[CR23] Office of Population Censuses and Surveys (1979). Census 1971 England and Wales general report part 1 definitions.

[CR24] Office of Population Censuses and Surveys (1981). Census 1981 definitions Great Britain.

[CR25] ONS. (2004). Census 2001 Definitions. London: The Stationary Office, Office for National Statistics/General Register Office for Scotland/Northern Ireland Statistics and Research Agency.

[CR26] ONS. (2013). 2011 Census Glossary of Terms. http://www.ons.gov.uk/ons/guide-method/census/2011/census-data/2011-census-user-guide/glossary/index.html. Accessed 15 October 2013.

[CR27] OPCS. (1992). 1991 Census Definitions Great Britain. London: Her Majesty’s Stationary Office/Office of Population Censuses and Surveys/General Register Office for Scotland.

[CR28] Poulain, M., Perrin, N., & Singleton, A. (2006). *THESIM: Towards harmonised European statistics on international migration*: Presses univ. de Louvain.

[CR29] Prioux F, Mazuy M, Barbieri M (2011). Recent demographic developments in France: Fewer adults live with a partner. Population (English Edition).

[CR30] Randall S, Coast E, Antoine P, Compaore N, Fatou-Binetou D, Fanghanel A (2015). UN census “Households” and local interpretations in Africa since independence. SAGE Open.

[CR31] Randall S, Coast E, Leone T (2011). Cultural constructions of the concept of household in sample surveys. Population Studies.

[CR32] Rault W (2012). De la rencontre à la vie commune. Quelques changements et continuités dans la formation des couples. Cahiers Francais.

[CR33] Régnier-Loilier, A., Beaujouan, É., & Villeneuve-Gokalp, C. (2009). Neither single, nor in a couple: A study of living apart together in France. *Demographic Research, 21*.

[CR34] Rowland DT (2003). Demographic methods and concepts.

[CR35] Schweber L (2006). Disciplining statistics: Demography and Vital Statistics in France and England 1830–1885.

[CR36] Smallwood S, Jefferies J (2003). Family building intentions in England and Wales: trends, outcomes and interpretations. Population Trends.

[CR37] Stillwell J, Coast E, Kneale D (2009). Fertility, living arrangements, care and mobility: Understanding population trends and processes (Vol. 1, UPTAP).

[CR38] Stone J, Berrington A, Falkingham J (2014). Gender, turning-points and boomerangs: returning home in the UK. Demography.

[CR39] THESIM (2006). http://www.uclouvain.be/cps/ucl/doc/gedap/documents/Poster_THESIM.pdf. Accessed 30 October 2013.

[CR40] UN (1959). *Handbook of population census methods: Volume III*—*Demographic and social characteristics of the population*. New York: Statistical Office of the United Nations.

[CR41] UN (1980). Principles and recommendations for population and housing censuses.

[CR42] UNSD (1997). Principles and recommendations for population and housing censuses revision 1.

[CR43] UNSD (2013a). Progression of the 2010 Census Round. http://unstats.un.org/unsd/demographic/sources/census/2010_PHC/censusclockmore.htm. Accessed 30 October 2013.

[CR44] UNSD (2013b). Census Round 2010: Progression of population censuses and the size of the enumerated population. http://unstats.un.org/unsd/demographic/sources/census/2010_PHC/Census_Clock/rptEnumeratedPopulationAllCountries.pdf. Accessed 15 October 2013.

[CR45] UNSD (2013). Overview of national experiences for population and housing censuses of the 2010 round.

[CR46] Valente, P. (2010). Census taking in Europe: how are populations counted in 2010? *Bulletin Mensuel d'Information de L'Institut National d'Études Demographiques, Population and Societies, 467*. http://www.unece.org/fileadmin/DAM/publications/oes/STATS_population.societies.pdf.

